# Who to Interview? Low Adherence by U.S. Medical Schools to Medical Student Performance Evaluation Format Makes Resident Selection Difficult

**DOI:** 10.5811/westjem.2016.10.32233

**Published:** 2016-11-29

**Authors:** Megan Boysen-Osborn, Justin Yanuck, James Mattson, Shannon Toohey, Alisa Wray, Warren Wiechmann, Shadi Lahham, Mark I. Langdorf

**Affiliations:** * University of California, Irvine, Department of Emergency Medicine, Irvine, California; † New York Presbyterian Hospital, New York, New York

## Abstract

**Introduction:**

The Medical Student Performance Evaluation (MSPE) appendices provide a program director with comparative performance for a student’s academic and professional attributes, but they are frequently absent or incomplete.

**Methods:**

We reviewed MSPEs from applicants to our emergency medicine residency program from 134 of 136 (99%) U.S. allopathic medical schools, over two application cycles (2012–13, 2014–15). We determined the degree of compliance with each of the five recommended MSPE appendices.

**Results:**

Only three (2%) medical schools were compliant with all five appendices. The medical school information page (MSIP, appendix E) was present most commonly (85%), followed by comparative clerkship performance (appendix B, 82%), overall performance (appendix D, 59%), preclinical performance (appendix A, 57%), and professional attributes (appendix C, 18%). Few schools (7%) provided student-specific, comparative professionalism assessments.

**Conclusion:**

Medical schools inconsistently provide graphic, comparative data for their students in the MSPE. Although program directors (PD) value evidence of an applicant’s professionalism when selecting residents, medical schools rarely provide such useful, comparative professionalism data in their MSPEs. As PDs seek to evaluate applicants based on academic performance and professionalism, rather than standardized testing alone, medical schools must make MSPEs more consistent, objective, and comparative.

## INTRODUCTION

The Medical Student Performance Evaluation (MSPE), formerly the “Dean’s Letter,” is a critical part of a medical student’s application to residency. The Association of American Medical Colleges (AAMC) guidelines, released in 1989 and updated in 2002 and 2016, emphasize that the document is an evaluation.^1^,^2^ Specifically, the MSPE should provide “an assessment of academic performance and professional attributes” that is “comparative, relative to [the student’s] peers.”^1^

According to the 2002 MSPE guidelines,^1^ the body of an MSPE highlights the student’s unique characteristics and narrative performance in basic sciences and clerkships, but it is difficult to extract tangible information from these sections to rank or judge the candidates.^3^ The appendices are meant to provide a program director (PD) with a “graphic representation of the student’s performance, relative to his/her peers,” in areas of pre-clinical courses, clerkships, professional attributes, and overall performance (appendices A, B, C, and D respectively).^1^ Appendix E is the medical school information page (MSIP) and contains essential information about the school’s assessment methods and compliance with various standards. The MSPE appendices enable a PD to evaluate a candidate’s academic performance during medical school, because grading policies are variable across United States (U.S.) medical schools.^4^ On September 29, 2016, the AAMC published updated guidelines for the MSPE, which now integrate the content of the appendices into the body of the MSPE.^5^

In general, MSPEs are written with inconsistent methods.^3^,^6^–^9^ While Shea and colleagues assessed frequency of the appendices as part of a larger work, 3 no studies have done a detailed evaluation of MSPE appendix variability. The purpose of this study is to determine each medical school’s compliance with the five recommended MSPE appendices, more than 10 years after the 2002 guidelines.^1^

## METHODS

We collected this data as part of another study that evaluated the MSPE ranking practices, but the methodology for the current study differed slightly as described below.^9^ We reviewed MSPE documents from applicants to the University of California, Irvine emergency medicine (EM) residency program in 2012–13 and 2014–15. We did not have the 2013–2014 application cycle documents electronically. We included MSPEs from U.S. allopathic medical schools, including Puerto Rico. We reviewed one MSPE per institution for each application cycle, according to which name appeared first alphabetically in the Electronic Residency Application Service (ERAS). We reviewed an MSPE from the University of California, Irvine internal medicine (IM) residency program application files for schools for which we did not have a 2014–15 MSPE. After this, we contacted the associate dean for student affairs from any school for which we did not have an MSPE.

A non-blinded, trained, single reviewer (JM or MBO) reviewed the MSPE in its entirety and completed a data abstraction form for the 2012–13 application cycle. A second, trained reviewer (JY or MBO) recorded data from the 2014–15 cycle on the same form. If data differed between years, we rechecked documents to ensure proper recording and used the practice pattern in the 2014–15 cycle for analysis. Just prior to data analysis, the primary study author (MBO) re-reviewed all data to ensure accuracy.

We reviewed the MSPE and recorded the following: 1) if the required information was in the appendix or elsewhere in the MSPE; 2) if the appendix was appropriately labeled; 3) if the student’s performance was specifically noted on the appendix; 4) if each of the 10 suggested MSIP elements was present. These MSIP elements are listed in supplementary Addendum 1 and described in *A Guide to the Preparation of the Medical Student Performance Evaluation*.^1^ The emphases, strengths, mission, and goals of the medical school were frequently indistinguishable from unusual characteristics of the educational program, so we counted these as one item. We also recorded whether the school used a pass/fail grading system, without the possibility of honors, or other equivalent two-tier grading system in the basic sciences and clerkships.

To meet criteria for professional attributes (Appendix C) the school needed a separate appendix discussing the school’s professionalism assessment or directing the reader to another area of the MSPE. Schools that mentioned generalities about their professionalism assessment in their MSIP did not meet criteria for the professional attributes appendix.

To qualify as an MSIP, the school needed an appendix that mentioned at least one of the 10 suggested MSIP elements (e.g., average length of enrollment). We did not include cover letters, unless they were labeled as a “Medical Student Information Page,” but we did mention the number of non-MSIP cover letters in our results.

To ensure that there was no variation between the IM MSPEs and the EM MSPEs, the primary study author (MBO) reviewed a portion (20% of the sample size) of IM MSPEs and calculated Cohen’s unweighted kappa.^10^ As a final measure of quality, the senior author (ML) reviewed a portion (20% of the sample size) of the EM study sample and calculated Cohen’s unweighted kappa.^10^

We calculated descriptive statistics for each question. The University of California, Irvine and the University of Illinois, Chicago, human subjects institutional review boards approved this study.

## RESULTS

### Subjects Enrolled

There were 136 U.S. allopathic medical schools with graduating classes in 2015; there were 132 in 2013.^11^ For each application cycle, our EM program receives approximately 650 applications and our IM program receives 2,000. We analyzed MSPEs from 134 of the 136 (99%) U.S. allopathic medical schools. We had MSPEs for both application cycles (2012–13 and 2014–15) for 114 (85%) of these medical schools; we had only the 2012–13 MSPEs for one school (1%) and 2014–15 MSPEs for 19 schools (14%).^9^ We reviewed 27 charts from the IM program to measure correlation; kappa was greater than 0.83 for all study questions and was equal to 1.00 for most (16/26 questions). Kappa for correlation between reviewers was greater than 0.86 for all questions and was equal to 1.00 for most (15/26 questions).

### Pre-clinical Performance (Appendix A)

Seventy-six (57%) schools had an appendix with comparative data for preclinical performance (Table 1) and four had the information in the MSPE body or a transcript. Forty-six (34%) were appropriately labeled as Appendix A. Many (n=51, 38% of total MSPEs) indicated the student’s performance on the graph (e.g., bolding, arrows). Of the schools that did not provide comparative preclinical data in an appendix or MSPE body, 32 of 54 (59%) used a pass/fail or other two-tiered grading system (i.e. a system that could not provide comparative data). For all parts of Appendix A, 29 (22%) schools were fully compliant, having an “Appendix A” with comparative preclinical data in graph or chart form, indicating the student’s performance on the graph.

### Clinical clerkship Performance (Appendix B)

One hundred and twelve (82%) schools had graphic comparative data for the clerkships in the appendix and eight (6%) had this information in the body of the MSPE (Table 1). Two schools without comparative clerkship data used a two-tiered grading system.

### Professional attributes (Appendix C)

Twenty-four schools (18%, Table 1) had a professional attributes appendix and three (2%) had a similar professionalism section in the body of the MSPE. Table 2 categorizes how each school provided their professionalism assessments. Only 10 of these assessments (7% of total MSPEs) were both specific to the student and comparative to the class. The figure provides examples of specific, comparative professionalism assessments from representative U.S. medical schools. The following were some of the professionalism behaviors assessed or mentioned in the MSPE appendices: time-keeping, preparedness for activities, teamwork, appearance, respect, compassion, reliability, interprofessional relationships, altruism, honesty/integrity, response to feedback, patient interactions, responsibility, pursuit of excellence, medical ethics, confidentiality, punctuality, self-confidence, verbal and written communication.

### Overall Performance (Appendix D)

Seventy-nine schools (59%) had information on overall comparative performance in their appendices (Table 1). This is not to be confused with the number of medical schools that provided comparative performance or rank (n=101, 75%) for their students at any point in the MSPE (for example, stating their student is in the “second quartile,” but not depicting the comparative performance in an appendix), which we report in a separate study.^9^

### Medical Student Information Page (Appendix E)

One hundred and fourteen schools (85%) had an MSIP (Table 1). The majority of medical schools had at least seven of the 10 MSIP elements (n=103, 77%) and more than half had all 10 (n=76, 58%). (See supplementary addendum 1.) Among schools without an MSIP (n=20, 15%), eight had an opening cover letter, but only one of these had at least seven of the suggested MSIP elements.

### Overall Compliance by Medical Schools with the Appendices

Twelve schools (9%) had five appendices present and 59 (44%) had four of five, not necessarily labeled correctly. Three schools (2%) were fully compliant with all appendices, having each one appropriately labeled, graphic, comparative, and student-specific; however, one of these schools was missing one of 10 MSIP elements.

### Grading Systems

Overall, 42 (31%) medical schools use a two-tiered grading system (e.g. pass/fail) for the basic sciences and two (1%) use one for the clinical clerkships.

## DISCUSSION

Despite the 2002 AAMC recommendations for better standardizations among the MSPE, there is still considerable variation. 3,6–9 The MSPE is the only comprehensive description of a student’s academic performance, personal qualities, and professionalism. Threats to the validity of the document, through inconsistency and lack of objectivity, compromise the value of the document in residency admissions. While only 2% of medical schools were fully compliant with all five appendices, most schools complied with at least one. Furthermore, most schools provided an MSIP and more than half of these had every necessary element. This suggests that student affairs officers are aware of the AAMC guidelines but have not modified their processes to comply.

It is unclear why medical schools do not comply with the MSPE guidelines. Some possibilities are that medical schools want a PD to read the MSPE in its entirety, not focusing on comparative data alone. Student affairs officers may fear that students will not successfully match if the student’s comparative data falls below the class mean. Furthermore, schools may not want to provide both positive and negative information for students, unless every medical school agrees to do the same.

PDs, however, must have some basis to judge candidates. Grade distributions vary tremendously between schools, with the number of students receiving an honors or equivalent top grade in the clinical clerkships ranging from 2–87% in one study.^4^ Furthermore, “honors” is a second-best grade at some schools.^4^ When the appendices are not present, a PD may find it difficult to extract concrete, comparative information from the MSPE. It is our opinion that narrative comments in the body of the MSPE are near-uniformly positive with little information to differentiate students. Without the appendices, a PD is unable to judge an applicant’s academic performance with respect to other candidates.^4^

Another possibility for lack of comparative performance is a two-tiered grading system (e.g. pass/fail), which inherently hinders discrimination in performance. We found that 41% of schools without comparative preclinical performance and 92% of schools without comparative clinical performance use a three- or more-tiered grading system (e.g. honors/pass/fail). Therefore, these schools do differentiate among their students in their internal grading system, but do not provide a legend to interpret this system to PDs.

While it is straightforward to provide comparative information for grades, extracting objective data for qualities such as professionalism can be more difficult. In our study, only a minority of schools provided a professionalism appendix and fewer were student-specific and comparative. Understandably, it may be difficult for schools to provide a comparative professionalism assessment for each student, since the majority of U.S. medical students should meet or exceed expectations in this area. However, it would be useful to highlight students who stray from the mean positively or negatively, since PDs value this information.^12^ A professionalism assessment tool was developed by the AAMC in 2005 (and is used by one school in our study), but this tool was never widely distributed and is no longer available online.^13^ The 2002 MSPE guidelines depict a histogram for the professionalism attributes appendix,^1^ but few schools provided this. Of schools that did provide specific, comparative professionalism assessments, most provided the student’s Likert scale score for one or more professional behaviors, compared to a class mean and standard deviation; these scores were commonly derived from assessments during the clinical clerkships. It is our opinion that this is a best practice for professionalism assessments on the MSPE.

There are many implications to the observations in this study. First, inconsistency in the MSPE decreases its value to PDs. As the MSPE is devalued, so is overall academic performance and professionalism, since the MSPE is largely the source for this information. As a result, PDs may overemphasize more objective data, such as United States Medical Licensing Examination scores, which could have negative consequences on medical education.^14^ Second, difficulty in interpreting the MSPE adds time to the already arduous job of screening over 800 applications each year.^12^ This takes a PD’s time away from other important aspects of the residency selection process, as well as from curriculum development and program administration. Furthermore, it undermines the time spent by all parties in the composition of the MSPE.

It is crucial that medical schools and the AAMC act to preserve the value of the MSPE by increasing its objectivity, consistency, and usability. Lack of comparative, student-specific assessments from the MSPEs does not force PDs to consider the entire document. Rather, it hinders the PD’s ability to compare applicants during the residency selection process. As a result, many of the qualities described in the MSPE are lost and may lead to overemphasis on standardized test scores. The 2016 AAMC’s MSPE guidelines emphasize the importance of graphic, comparative information regarding students’ academic performance.^5^ Comparative clerkship performance should now be integrated into the body of the MSPE. Comparative overall performance and comparative performance in the core competencies should now be included in the summary. These revised guidelines must be introduced systematically, with medical schools being held accountable for compliance with them. We recommend that future guidelines provide clear instructions on how medical schools should assess overall professional attributes.

## LIMITATIONS

This study relied on a convenience sample of MSPEs to our EM and IM residency programs, but our sample reflected 99% of U.S. allopathic medical schools. We analyzed one document per school per application year, but we minimized this limitation by analyzing two application years and comparing a portion of MSPEs between two specialties. We did not determine the degree to which the MSPE affected candidate interview or ranking.

## CONCLUSION

The content of MSPE appendices (now within the body and summary of the MSPE) are designed to provide PDs with graphic, comparative, student-specific information regarding academic performance and professionalism. Medical schools have low overall compliance with the appendices, most notably in the professional attributes Appendix C. Low compliance in providing graphic, comparative performance information among medical schools decreases a PD’s ability to use the MSPE to compare candidates.

## Supplementary Information



## Figures and Tables

**Figure:**
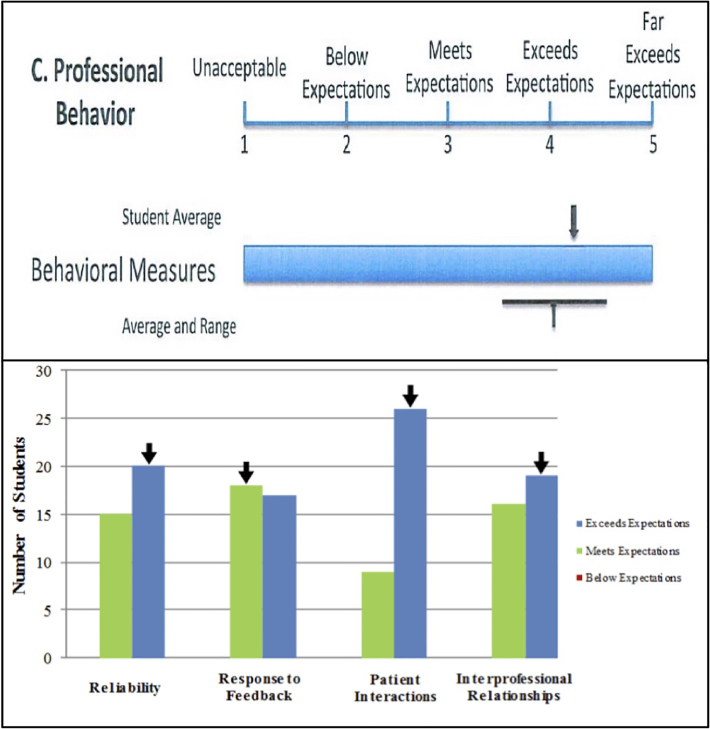
Representative professionalism assessment from two U.S. medical schools.

**Table 1:** Degree of compliance with each of the recommended medical student performance evaluation (MSPE) appendix items among U.S. medical schools (n = 134)

Number (percent) of schools that had the following information:	Appendix A pre-clinical performance	Appendix B clerkship performance	Appendix C professional attributes	Appendix D overall performance	Appendix E med school info page (MSIP)
Had the information present in the appendices	76 (57%)	112 (82%)	24 (18%)	79^c^ (59%)	114 (85%)
Had appendix present and it was appropriately labeled	46 (34%)	50 (37%)	22 (16%)	37^c^ (28%)	68 (51%)
Information presented in graphic form (e.g. bar graph)	68 (51%)	102 (76%)	6 (6%)	46 (34%)	N/A
Schools that indicated the student’s performance on the appendix	51 (38%)	67 (50%)	11^a^ (8%)	32 (24%)	N/A
Information found elsewhere in the MSPE or in the transcript	4 (3%)	8 (6%)	3^b^ (2%)	11^c^,^d^ (8%)	8^e^ (6%)
Schools that indicated that the data could not be provided in the respective appendix	12 (9%)	2 (1%)	3 (2%)	2 (2%)	0 (0%)
Schools fully compliant with this appendix (appropriately labeled + comparative + in graphic form + student-specific)	29 (22%)	34 (25%)	5 (4%)	19 (14%)	54^f^ (40%)

a Some of these only mentioned that the student met the professionalism standards for the school, without other specific data.

b Found in the MSPE in a professionalism section or graph

c These particular values are similar and related to results for a separate related study that looked at different features of the MSPE (ranking methods).^9^ The number for the first and second row is larger in this study than in the previously published study,^9^ accounting for schools that had an appendix present that directed the reader to a part of the MSPE which contained the class rank, but did not fully explain the ranking system in appendix D.

d Six were found in a cover letter and five were found in the body of the MSPE.

e This number represents schools who had an opening cover letter that was not labeled as a medical student information page.

d Fully compliant for appendix E means that the MSIP contained 10 of 10 MSIP elements and was appropriately labeled.

**Table 2:** Description of professionalism assessments used in U.S. medical schools’ medical school performance evaluations (MSPE) (includes those found in appendix C or the MSPE body, n = 27).

Summary of professionalism assessment in MSPE	n = (% of 27)
1. Refers reader to the MSPE clerkship narratives or summary paragraph	10 (37%)
2. Refers reader to the MSPE, which contains a professionalism score	3^a^ (11%)
3. Provides Likert score for professionalism behavior(s), without comparative class data	2 (7%)
4. Provides Likert score for professionalism behavior(s), with a class mean^b^	7 (26%)
5. Describes the school’s general assessment methods and states that the student met those expectations or gives a brief qualitative description of the student’s professional behaviors	5^c^ (19%)

a One school has a professionalism distinction for the top students only.

b The authors feel this is a best practice.

c One of these did not have a sentence stating that the student met those expectations.
