# Supplementary material for: Who to Interview? Low Adherence by U.S. Medical Schools to Medical Student Performance Evaluation Format Makes Resident Selection Difficult
Source: West J Emerg Med. 2016 Nov 29;18(1):50–5. doi: 10.5811/westjem.2016.10.32233 (PMC5226763; doi:10.5811/westjem.2016.10.32233)
Supplement: Supplementary file 1 [file wjem-18-50-s001.docx]

Supplementary Addendum 1: Compliance with the 10 medical student information page (MSIP) elements, among schools with an MSIP (n=114):

| MSIP contains the following: | Number of schools (%) |
| --- | --- |
| 1, 2. Program emphases, strengths, mission, goals, unusual characteristics^a^ | 110 (96%) |
| 3. Average length of enrollment | 95 (83%) |
| 4. Compliance with AAMC academic transcript guidelines | 95 (83%) |
| 5. Description of the evaluation system | 107 (94%) |
| 6. USMLE requirements | 112 (98%) |
| 7. OSCE use | 106 (93%) |
| 8. Use of narrative comments for MSPE | 96 (84%) |
| 9. Process by which the MSPE is composed | 103 (90%) |
| 10. Information about whether the student is permitted to review the MSPE | 102 (89%) |
| Schools compliant with at least seven of the MSIP elements | 103 (90%, 77% of total^b^) |
| Schools compliant with all 10 elements of the MSIP | 76 (66%, 58% of total^b^) |
| Schools compliant with all 10 elements AND appendix is labeled correctly (Appendix E) | 54 (47%, 40% of total^b^) |

Abbreviations: MSIP: Medical School Information Page, AAMC: Association of American Medical Colleges, MSPE: Medical Student Performance Evaluation, USMLE: United States Medical Licensing Examination, OSCE: Objective Structured Clinical Examination

a: For a detailed description of each element, see: AAMC: A Guide to the Preparation of the Medical Student Performance Evaluation.^1^ The first two elements are grouped together for analysis.

b: These values are the percentage among schools that had an MSIP (n=114) and percentage among total medical schools studied (n=134), respectively.
